# An Investigation of the Dementia Care Competences and Related Factors of the Dementia Case Manager

**DOI:** 10.1002/alz70858_099225

**Published:** 2025-12-25

**Authors:** Shu‐Jun Zhang, Huei‐Ling Huang

**Affiliations:** ^1^ Chang Gung University of Science and Technology, Taoyuan City, Guishan Dist., Taoyuan City, Taiwan; ^2^ Chang Gung University of Science and Technology, Taoyuan, Taiwan

## Abstract

**Background:**

This study aimed to investigate the dementia caregiving competence of case managers and related factors. Few studies have been conducted on the caregiving competence of case managers. The results of this study will serve as a reference for training and managing dementia case management professionals, ultimately improving care quality, effectiveness, and reducing caregiver burden.

**Research Methods:**

This study used part of the data from Dr. Huang's National Science and Technology Council (NSTC) grant project: “Establishment of Core Competence and Training Models for Dementia Case Management” to conduct the secondary data analysis. Descriptive correlation study was adopted with convenience sampling and 143 dementia case managers were involved in the study. The study recruited participants from August 2019 to December 2022. After obtaining informed consent, data was collected. Data analysis was performed using descriptive statistics, independent sample t‐tests, one‐way ANOVA, Pearson correlation, and multiple regression.

**Results:**

On average, dementia case managers scored 75.6 out of 100 on dementia care professional competence, indicating a moderate level of competency. Key factors influencing overall dementia care competence include current service experience and years of service, previous experience of dementia care, having received dementia‐related training within two years, having received basic/advanced training from the Ministry of Health and Welfare, trained as a case manager/health educator, willingness to care, and self‐confidence in caring for dementia. Among them, “having received a case manager/health educator training course and willingness to care for persons with dementia” was the most important predictor of dementia care competence among case managers, with an overall explained variance of 11.9%.

**Discussion:**

This study highlights disparities in dementia case managers' core competencies across nine key subcategories. These findings can be used as a reference for the training of dementia case managers in clinical care practice and education.

